# Explanatory Capacity of Postural Control and Physical Fitness in Cognitive Impairment and Support Needs among Individuals with Intellectual Disabilities—A Cross-Sectional Pilot Study

**DOI:** 10.3390/brainsci13081213

**Published:** 2023-08-17

**Authors:** María Mercedes Reguera-García, Eva Fernández-Baró, Ignacio Diez-Vega, Irene Varona-Echave, Jesús Seco-Calvo

**Affiliations:** 1 SALBIS Research Group, Department of Nursing and Physiotherapy, Faculty of Health Sciences, University of León, 24400 Ponferrada, Spain; 2 Asprona Bierzo, Ave 3rd 24 A. Compostilla, 24402 Ponferrada, Spain; 3 ENSADE Research Group, Department of Nursing and Physiotherapy, Faculty of Health Sciences, University of León, 24400 Ponferrada, Spain; 4 Exercise, Health and Applied Biomarkers Research Group, European University of Madrid, 28670 Madrid, Spain; 5 Clínica Volta do Castro, Rúa de Feliciano Barrera Fernández, 9, BAJO, 15706 Santiago de Compostela, Spain; 6 Institute of Biomedicine (BIOMED), University of León, 24071 León, Spain; 7 Physiology Department, University of the Basque Country, 48940 Leioa, Spain

**Keywords:** Mini BESTest, Senior Fitness Test, anticipatory postural adjustments, consecutive postural adjustments, motor strategies, Arm Curl Test

## Abstract

Postural control is a skill associated with most motor activities and is essential for the performance of activities of daily living. People with intellectual disabilities (ID) present postural control deficits that can be attributed to several causes. The aim of this study was to determine whether postural control and physical fitness could explain the cognitive impairment and support needs in this population. A cross-sectional pilot study was conducted with 18 people with ID. Data collection was based on assessments for postural control (Mini BESTest and Berg Balance Scale) and physical fitness (Senior Fitness Test). The data were analyzed using linear regression models. Anticipatory postural adjustments were associated with support needs, explaining up to 45% of these. Consecutive postural adjustments and upper limb strength were less significantly associated with support needs. However, none of the variables used explained cognitive impairment in ID. Knowledge of the relationships and behavior of the different measurement tools is essential for the development of appropriate interventions in this population.

## 1. Introduction

Intellectual disability (ID) is a neurodevelopmental disorder characterized by significant limitations in intellectual functioning and adaptive behavior, identified before the age of 22 years [1,2]. People with ID have difficulties in reasoning, problem solving, planning, understanding abstract concepts and learning. In addition, they have deficits in other areas such as communication, self-care or personal relationships; therefore, they require support in different aspects of daily life for better individual functioning [3,4]. Other characteristics associated with ID include delays in the development of motor skills, as described in the reviews consulted [5,6].

Among the motor skills that may be altered in ID, postural control is observed, especially if it is affected in the central nervous system or in motor development during childhood or adolescence [7,8]. Postural control refers to the ability to maintain control of position in space for the dual purpose of stability and orientation. Postural stability is the ability to control the center of mass in relation to the base of support. And, on the other hand, orientation is the ability to maintain an adequate relationship between the segments of the body and the environment to perform a specific task such as walking. Postural control also requires cognitive resources such as attention and learning [9]. Assessments of postural control are manifold. Among these assessments, the functional capacity measures developed initially for the elderly and later validated in ID, such as the Berg Scale to observe the risk of falling, stand out [10]. Or measurements aimed at differentiating underlying systems such as postural adjustments, sensory orientation and dynamic gait [11]. Postural adjustments are motor strategies to external perturbations, activated before, during or after movement. Simulated postural adjustments (SPA) are activated during movement and involve greater complexity because the postural component and the focal component must be studied kinematically. Anticipatory postural adjustments (APA) are muscular activities prior to an intentional and predictable external motor action [12]. Reactive or consecutive postural adjustments (CPA) are muscular activities that follow an actual alteration of postural control [13]. These last two automatic postural responses can be easily analyzed with the BESTest [14] Sensory orientation is the ability to coordinate body parts with respect to the base of support, gravity, visual environment and internal references. Dynamic gait is gait in different situations or tasks. Physical characteristics of Down syndrome such as muscular hypotonia, severe obesity in Prader–Willi syndrome, or poor coordination in the upper and lower extremities seen in autism spectrum disorder, justify the importance of studying postural control deficits in this population [15,16]. Recent literature reviews on ID interventions note that postural control is impaired in both stability and orientation [7]. Most studies on ID have focused on analyzing gait orientation, observing altered gait patterns, delayed responses to disturbances and an increased number of falls [17,18]. And there is increasing interest in the postural strategies of the systems involved [5,19].

Most definitions of physical fitness consider physical fitness, which includes body composition, cardiorespiratory endurance, muscular strength and endurance, and flexibility. In addition, physical fitness, in its broadest sense, should also include motor fitness, which includes speed, postural control, coordination and mobility, among others [20] as essential components for the performance of activities of daily living and the development of functional skills to respond to the demands of the environment more efficiently [21,22,23].

Some studies with adults show that certain physical conditions such as cardiorespiratory endurance or the strength of the prehensile muscles are related to cognition [24,25]. In fact, some physical functions, such as walking speed, may begin to decline simultaneously with, or even before, the decline in cognitive function. The degree of lower limb function has also been associated with the risk of developing Alzheimer’s disease [19]. Different anatomical and physiological features, such as reduced brain size, decreased neurotransmitter and neuronal density, abnormalities in myelination, alterations in muscle properties or sensory deficits, are characteristic of people with ID [7]. Alterations in cognitive functions, the presence of premature ageing and a sedentary lifestyle could also be added as causes of the problems experienced by this population in postural control and lower levels of performance and decreased physical abilities [6,21]. Young adults with ID have 8–12% lower cardiorespiratory fitness. Elbow and knee flexion and extension muscle strength in young adults with ID showed 35–40% lower levels than sedentary individuals without disability [26]. New findings suggest that people with ID are at increased risk of health problems compared to the general population [21,22]. People with ID experience an earlier ageing process, starting around 40–50 years, are more likely to have an unhealthy life expectancy and greater cognitive decline [10,20,22]. The Senior Fitness Test (SFT) presents an accessible level of difficulty for people with ID and conveys confidence when performing the tests. Ayaso-Maneiro et al. [27] used the SFT to assess physical fitness in adults with ID with a mean age of 42 years. The deterioration of physical skills associated with the onset of premature aging and other comorbidities such as osteoporosis, diabetes or musculoskeletal disorders convert adults with ID with physical fitness levels that could be compared with the older population [22]. Due to the ID situation, support needs can help people with ID to overcome barriers and to improve and reach their maximum potential in adaptive behavior. Adaptive behavior is essential for independence and autonomy in daily life. There is a categorization based on intermittent, limited, extensive and generalized support needs [4].

Given these cognitive impairment and support needs, it is necessary to examine levels of postural control and physical fitness to understand how this population functions and to prevent negative consequences, such as chronic illness, falls and loss of independence [10,17]. A better understanding of these issues could lead to the development of more appropriate strategies to address these limitations that affect the daily functioning of people with ID. Therefore, the aim of this study was to assess the explanatory power of postural control and physical fitness in determining cognitive impairment and support needs in people with ID.

## 2. Materials and Methods

### 2.1. Study Design

A descriptive, cross-sectional pilot study was carried out. Participants were selected by non-probabilistic convenience sampling.

### 2.2. Description of the Sample

Due to the paucity of studies with similar characteristics, we decided to use pilot data to estimate the effect size. An effect size (r^2^) between 0.3 and 0.4, a probability of error α = 0.05, a power 1 − β = 0.8 and a number of predictors between 11 and 12 were considered. Two simulations were performed through multiple linear regression models: one with r^2^ = 0.3 and 12 predictors and another with r^2^ = 0.4 and 11 predictors. With these simulations, a total of between 37 and 52 participants would be necessary, which is why this was considered a pilot study.

The selected participants belonged to the day center service of an association of people with ID. There were 27 users of this service, all of whom were invited to participate. The inclusion criteria were as follows: (a) being of legal age; (b) mild–moderate intellectual disability with a score of 60–90 points in the General Functionality item of the Inventory for Client and Agency Planning (“ICAP”); (c) autonomous ambulation; (d) ability to maintain the standing position. The exclusion criteria consisted of the following: (a) the use of walking devices and (b) current pathology of the musculoskeletal system or pathologies with alterations in postural control.

The participants or their legal guardians were previously informed of the objectives of the study. If they agreed to participate, in accordance with the Declaration of Helsinki (rev. 2013), all participants or signed an informed consent prior to their participation in the study. The institutional review board approved the study protocol and granted the ethical approval from the Ethics Committee of the University of León (code: ÉTICA-ULE-047-2022).

### 2.3. Procedure

Participants were contacted and summoned for an initial assessment on two separate days, 24–48 h apart. On the first day, sociodemographic descriptor variables (age, weight, height), cognitive impairment (Mini Mental State Examination (“MMSE”) [28]), an assessment of the need of support services for adaptive and maladaptive behaviors (“ICAP”) [29] and postural control variables such as the Berg Balance Scale (“BBS”) [10], Functional Reach Test (“FRT”) [30], Timed Up and Go Test (“TUG”) [6] and the Six Spot Step Test (“SSST”) [30] were recorded. On the second day, physical fitness variables (Senior Fitness Test (“SFT”) [31]) and postural control variables (Mini BESTest (“MBT”) [11]) were recorded. For tests performed on the same day, a five-minute rest period was given between tests. The tests were administered by two physical therapists previously trained in the evaluation protocol.

Cognitive impairment was assessed using the MMSE. This measure was adapted and validated in Spanish by Lobo in 1975 [28]. It encompasses seven areas of cognitive functioning. The MMSE includes items assessing orientation, memory and attention, as well as items requiring language skills, writing and following commands [28]. It has a good test–retest reliability (0.80–0.95) and acceptable sensitivity to detect mild to moderate stages of dementia [32].

ICAP is a scale designed for planning and evaluating services for people with ID using a nine-level rating system. The results of the different subscales (adaptive and maladaptive behavior) form a combined score that indicates the need for attention, supervision, and training. Adaptive behavior includes assessment of motor, social and communication skills, as well as daily living and community skills [26]. The adaptive and maladaptive behaviors and service needs of individuals with developmental disabilities was assessed using the ICAP [29]. This measure consists of 77 items that include motor skills, personal living skills, community living skills, social and communication skills, general independence and behavior. The items of these dimensions are scored on a four category Likert-type scale. ICAP presented adequate reliability that ranged between 0.86 and 0.98 for people without disabilities and between 0.88 and 0.98 for people with disability [29,33].

Postural control was evaluated using the MBT and the BBS.

MBT [34] is a reduced test of the BESTest [11]. It evaluates 14 items. Four constructs and a total score are grouped together. The four constructs are as follows: (a) APA, (b) CPA, (c) sensory orientation and (d) dynamic gait. Anticipatory postural control assesses how to move from sitting to standing, standing on tiptoes and single leg standing. Reactive postural control assesses compensatory correction with a forward step, backward step and sidestep. Sensory orientation analyzes the ability to maintain balance on a stable/unstable/tilted surface with eyes open and closed. Dynamic gait analyzes changes in speed, horizontal head turns, pivot turns and obstacles while walking. The reliability of the MBT is excellent to good, with ICC values > 0.90 for people with various diagnoses. In addition, it has a high validity [35].

BBS is a functional scale that evaluates static and dynamic balance. It is based on 14 frequent activities of daily life. These tasks are scored between 0 and 4 points, with a total score of 56 points [36]. This scale is used to measure postural control in people with ID [32]. The relative intrarater reliability is excellent in older people (95% CI 0.97 to 0.99). The relative inter-rater reliability is also high (95% CI 0.96 to 0.98) [37].

Physical fitness was measured using SFT [31]. This is a widely used battery in healthy older people which evaluates several different variables: (a) lower limb strength, the Chair Stand Test, number of repetitions in one minute; (b) upper limb strength, the Arm Curl Test, number of repetitions performed in 30 s with a weight of 2.5 kg/women and 4 kg/men; (c) aerobic endurance, the 6 min walk test, meters walked; (d) lower limb flexion and flexibility, the chair sit and reach test, distance to the tip of the foot with the leg straight; (e) upper limb flexibility, the back scratch test, distance between the tips of the third toes of both hands; (f) dynamic balance, 8 foot up and go test, time to stand up, walk and sit down again at maximum speed. The reliability and validity of these tests have been studied [38]. In addition, we found stratified normative data in various populations, concretely, Americans [31], Turks [39] and Serbs [40].

### 2.4. Data Analysis

Frequencies and percentages were used to describe the results of the qualitative variables and means, whereas the standard deviation and range were used to describe the results of the quantitative variables.

The inferential analysis was carried out through linear regression analysis, using the scores from the MMSE and the ICAP as dependent variables. The result of MMSE, along with the MBT, BBS and SFT, were used as predictor variables for each of the models described. After verifying compliance with the parametric assumptions, four regression models were analyzed to identify the best explanatory variables. Models 1 to 3 were analyzed using the introduce method (model 1: bivariate; model 2: multivariate with the dimensions of each test; model 3: multivariate with all the dimensions of all the tests). Cognitive function was not associated with any explanatory variable (*p* > 0.05). The dimension that contributed the most in all the models was MBT-a.

In the event of finding statistically significant models, a fourth model was built using the stepwise method, automatically selecting the predictor variables with the best explanation in the model.

Standardized and non-standardized coefficients, 95% confidence intervals (95% CI), *p*-value, and adjusted coefficient of determination (r^2^) are reported. The significance level was set at *p* = 0.05.

## 3. Results

### 3.1. Participants Characteristics

**Figure 1:**
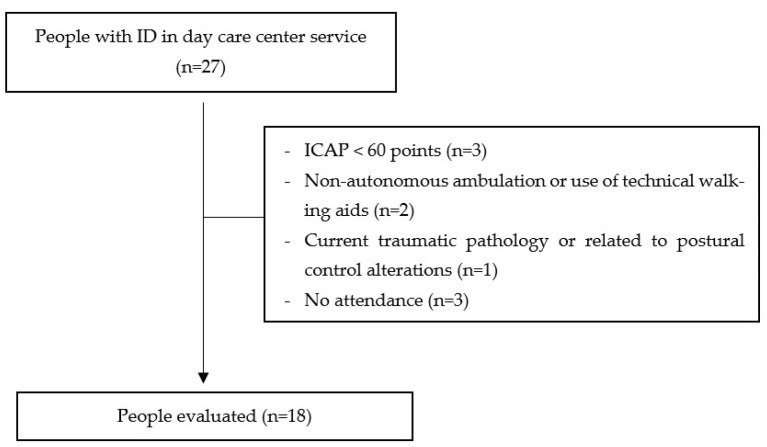
CONSORT flowchart diagram.

A total of 18 participants participated in the study (see Figure 1, CONSORT flowchart diagram). Table 1 presents the main characteristics of the study participants.

The sample consisted of 10 men and 8 women. The participants were adults (37.50 ± 10.75 years), overweight (28.63 ± 7.18 points in IMC), with mild cognitive impairment (22.28 ± 5.27 MMSE) and in need of limited care with periodic follow-up or without the need for assistance in daily living (7.33 ± 0.91 ICAP). In addition, they were functionally active with a low risk of falls.

### 3.2. Cognitive Impairment

Cognitive impairment was not associated with any explanatory variable (*p* > 0.05). The MBT-APA dimension was the one that best explained cognitive function, with a standardized coefficient β = 0.44 in model 1, a standardized coefficient β = 0.73 in model 2 and a standardized coefficient β = 1.42 in model 3 (Table 2).

### 3.3. Support Service Needs

Support service needs were associated with MBT-T, MBT-APA, MBT-CPA and SFT_2 (model 1: *p* < 0.05).

The MBT dimensions significantly explained the support needs for adaptive and maladaptive behaviors (model 2: F = 3.75 (4); *p* = 0.03; adjusted r^2^ = 0.39), again MBT-APA was the dimension that best explained the results (β = 0.71). Indeed, it was the only dimension identified in model 4, explaining 45% of the variance of support needs (model 4: F = 14.30 (1); *p* < 0.001; r^2^ = 0.

Although the SFT-2 dimension also demonstrated an association with support needs (model 1: F = 5.94 (1); *p* = 0.01; r^2^ = 0.27) it did not significantly contribute to explain support needs in the rest of the models (*p* > 0.05) (Table 3).

## 4. Discussion

The aim of this study was to determine whether the evaluated components of postural control and physical fitness could explain cognitive impairment and support needs in people with ID.

Although the components of postural control and physical fitness failed to explain cognitive impairment, they were able to explain the support needs of people with ID.

First, our results do not allow us to demonstrate the explanatory capacity of postural control (measured with MBT and BBS) and physical fitness (measured with the SFT) in cognitive impairment in people with ID. APA are the dimension that explains cognitive impairment the best. According to our literature search, no similar studies have been found with the same objective. We believe this is because people with ID evoke learned movements necessary for activities of daily living later [38]. Therefore, recent research in the physical and movement performance of people with ID employs the Mini BEST as a means of assessing various sub-systems of postural control in functional exercise interventions [7].

Healthcare professionals are concerned about the rehabilitation of people with cognitive impairment [41]. But the cognitive impairment assessed using the MMSE does not correspond to the conceptual skills included in the concept of adaptive behavior. Adaptive behavior is divided into conceptual (cognitive), social and practical (ADL) [26]. The MMSE is a gold standard for detecting cognitive impairment in the general population, especially in research studies [42]. However, MMSE has not been validated for use in ID, and there is limited information on its validity in this group. Although some researchers have used the MMSE in ID, others suggest that this test may have limited utility because of the difficulties in completing the assessment [43]. As expected, there is no relationship between reflex, autonomic, voluntary motor skills and cognitive skills in people with ID with mild impairment measured with the MMSE (orientation, calculation, recall and language). Some of the more demanding postural control factors (anticipatory) come close to explaining the cognitive impairment due to the multiple neural connections related to the performance of postural adjustments.

Secondly, the results related to the need for support services allow us to test the explanatory capacity of the total scores, APA and CPA, as well as of the upper limb strength of the SFT. No association was detected between BBS and MMSE and support needs.

APA were the dimension that best explained support needs (45%). We believe this is due to several factors. First, APA are based on the activation of SPA. These adjustments are motor changes in the body that provide prior postural stability (termed the “postural” component) [12] for the performance of changing motor tasks in stable and unstable environments. These motor changes or prior adjustments occur in most of the skills analyzed in the ICAP [44]. Second, most of the ICAP skills are motor skills developed in childhood, activities of daily living and instrumental activities essential for adapting to life in the community [29]. Therefore, activities of daily living require APA prior to the task to improve performance and execution, as well as autonomic control to cope with the demands of the environment. Finally, both the performance of ICAP skills and APA involve multiple structures of the nervous system such as the basal ganglia, cerebellum, premotor cortex, supplementary motor cortex and the primary motor cortex [45,46].The second dimension that provides a better estimation of support needs is the MBT total score. We believe that this is because the MBT comprises numerous motor tests necessary to improve motor learning of ICAP skills. In addition, it is worth noting that half of the dimensions are statistically related in model 1, and therefore, it is logical to assume that the total score of the test will have explanatory power.

The Arm Curl Test, which is upper limb strength measured using the SFT, is another dimension that explains the need for support services. Similarly, other investigators have demonstrated the importance of strength in ID [47]; lower muscle strength measured with a hand-held dynamometer is associated with a decrease in the performance of activities of daily living in both basic activities measured with the Barthel index and may lead to bone frailty [48]. Similarly, in older people, grip strength is a discriminatory measure of neurological function and brain health [49]. Likewise, several studies have identified grip strength as a strong predictor of nutritional status, muscle mass, physical function and health status, mortality and resource utilization [50].

The last explanatory dimension of support needs is reactive postural adjustments. This skill is essential as a last resort to avoid falls. In the case of people with pathological alterations of postural control, there is a delay in the onset of muscular activity related to postural adjustments when performing tasks. This situation requires the need for caregivers to supervise the person’s movements and/or needs [51].

It is striking that the BBS is not related to support needs. The BBS is a proposed balance assessment tool for ID [23] and commonly used to demonstrate the effects of functional balance programs [52]. Perhaps this is due to the low age of the sample, the predominance of males, and the fact that this sample had no associated visual impairment.

This study presents several methodological limitations. The results should be evaluated with caution because the sample size is limited, and with very specific characteristics. Also, the sample is not representative of the entire population with ID, especially those with more severe impairment. In addition, the methodology could not be complemented with three-dimensional (3-D) models or electromyography to be able to analyze the SPA (focal or postural) and APA.

One of the main strengths is the novelty of examining this specific population, which is usually not considered in research in the field of postural control and requires a large amount of material support resources.

Future lines of research should seek to perform treatments directed at improving postural control systems in ID, specifically APA. Furthermore, they should strive to improve the understanding of postural control systems with three-dimensional models of APA, SPA and CPA and support needs in ID. Our results identify those functional tests that are easy to access and understand, and provide valid information for assessing the support needs of people with ID. In addition, they can provide knowledge to professionals specialized in intellectual and motor disabilities, defining more appropriate strategies such as specific training programs focused on postural control that aim to improve functionality and quality of life.

## 5. Conclusions

The components of postural control and physical fitness provide insight into the support needs of people with ID, although they failed to explain their cognitive impairment. APA indicate the support needs and cognitive impairment. MBT total score, upper limb strength and CPA also explain the needs for support services in people with ID. However, no component of postural control or physical fitness can explain the findings regarding cognitive impairment.

## Figures and Tables

**Table 1:** Descriptive characteristics of the participants.

	Mean ± Sd	Range Min–Max
Age (years)	37.50 ± 10.75	22.00–58.00
Weight (kg)	76.83 ± 21.46	46.90–124.20
Height (cm)	163.67 ± 13.72	143.00–201.00
BMI (kg/m^2^)	28.63 ± 7.18	18.83–42.98
TUG (s)	7.29 ± 1.97	5.07–11.70
FRT (cm)	27.33 ± 10.92	17.00–64.00
SSST (s)	10.66 ± 2.89	7.29–18.42
MMSE (0–30 points)	22.28 ± 5.27	12.00–30.00
ICAP (score)	7.33 ± 0.91	6.00–9.00
MBT-T (0–28 points)	19.61 ± 4.65	11.00–27.00
MBT-APA (0–6 points)	3.78 ± 1.44	2.00–6.00
MBT-CPA (0–6 points)	3.06 ± 1.98	0.00–6.00
MBT-SO (0–6 points)	5.28 ± 1.07	3.00–6.00
MBT-G (0–10 points)	7.50 ± 1.58	4.00–10.00
BBS (0–56 points)	51.17 ± 3.96	43.00–56.00
SFT-1 (n rep)	11.83 ± 3.52	7.00–18.00
SFT-2 (n rep)	14.11 ± 4.03	7.00–21.00
SFT-3 (cm)	−24.24 ± 13.07	−42.00–0.00
SFT-4 (cm)	−23.22 ± 14.96	−47.00–0.00
SFT-5 (m)	265.43 ± 101.12	150.40–555.00
SFT-6 (s)	7.30 ± 1.98	5.07–11.70

BMI: body mass index; MMSE: Mini Mental State Examination; ICAP: Inventory for Client and Agency Planning; BBS: Berg Balance Scale; TUG: Timed Up and Go Test; FRT: Functional Reach Test; SSST: Six Spot Step Test; MBT-T: Mini BESTest total; MBT-APA: Mini BESTest anticipatory postural adjustments; MBT-CPA: Mini BESTest reactive or consecutive postural adjustments; MBT-SO: Mini BESTest sensory orientation; MBT-G: Mini BESTest gait; SFT-1: Chair stand test; SFT-2: Arm Curl Test; SFT-3: Chair sit and reach test; SFT-4: Back scratch test; SFT-5: 6 min walk test; SFT-6: 8 foot up and go test.

**Table 2:** Regression models to study the association of explanatory variables with cognitive impairment.

	Model 1					Model 2					Model 3				
	B	(95% CI)	β	*p*	r^2^	B	(95% CI)	β	*p*	r^2^	B	(95% CI)	β	*p*	r^2^
MBT-T	0.41	(−0.15; 0.97)	0.37	0.14	0.13									0.43	0.15
MBT-APA	1.63	(−0.11; 3.37)	0.44	0.07	0.20	2.68	(−0.19; 5.54)	0.73	0.23	0.13	5.17	(−1.57; 11.91)	1.42		
MBT-CPA	0.92	(−0.40; 2.24)	0.35	0.16	0.12	0.75	(−0.84; 2.34)	0.28			1.85	(−1.51; 5.22)	0.70		
MBT-SO	0.44	(−2.15; 3.03)	0.09	0.72	0.01	−1.98	(−5.22; 1.27)	−0.40			−4.31	(−12.37; 3.75)	−0.87		
MBT-G	0.58	(−1.16; 2.32)	0.17	0.49	0.03	−1.07	(−3.22; 1.14)	−0.32			0.21	(−3.59; 4.02)	0.06		
BBS	−0.02	(−0.72; 0.68)	−0.02	0.95	0.00						−1.40	(−3.05; 0.26)	−1.05		
SFT-1	0.48	(−0.27; 1.23)	0.32	0.20	0.10	0.62	(−0.93; 2.17)	0.42	0.83	−0.26	−0.92	(−3.98; 2.13)	−0.62		
SFT-2	0.47	(−0.17; 1.12)	0.36	0.14	0.13	0.20	(−0.84; 1.24)	0.15			0.61	(−0.67; 1.88)	0.46		
SFT-3	−0.01	(−0.24; 0.21)	−0.04	0.89	0.00	−0.04	(−0.41; 0.34)	−0.09			0.07	(−0.52; 0.67)	0.18		
SFT-4	0.05	(−0.14; 0.23)	0.13	0.61	0.02	0.07	(−0.20; 0.34)	0.19			0.05	(−0.31; 0.41)	0.13		
SFT-5	−0.01	(−0.03; 0.02)	−0.13	0.62	0.02	−0.01	(−0.05; 0.03)	−0.19			0.03	(−0.03; 0.09)	0.60		
SFT-6	−0.33	(−1.73; 1.07)	−0.13	0.62	0.02	0.40	(−2.45; 3.25)	0.15			−0.57	(−6.51; 5.36)	−0.22		

Dependent variable: MMSE. MMSE: Mini Mental State Examination; MBT-T: Mini BESTest total; MBT-APA: Mini BESTest anticipatory postural adjustments; MBT-CPA: Mini BESTest reactive or consecutive postural adjustments; MBT-SO: Mini BESTest sensory orientation; MBT-G: Mini BESTest gait; BBS: Berg Balance Scale; SFT-1: Chair Stand Test; SFT-2: Arm Curl Test; SFT-3: Chair sit and reach test; SFT-4: Back Scratch Test; SFT-5: 6 min walk test; SFT-6: 8 foot up and go test.

**Table 3:** Regression models to study the association of explanatory variables with support needs.

	Model 1					Model 2					Model 3					Model 4				
	B	(95% CI)	β	*p*	r^2^	B	(95% CI)	β	*p*	r^2^	B	(95% CI)	β	*p*	r^2^	B	(95% CI)	β	*p*	r^2^
MBT-T	0.13	(0.05; 0.21)	0.65	0.004	0.42										0.36					
MBT-APA	0.44	(0.20; 0.68)	0.69	0.001	0.48	0.45	(0.38; 0.86)	0.71	0.031	0.39	1.14	(−0.31; 2.59)	1.81			0.44	(0.19; 0.69)	0.70	0.002	0.45
MBT-CPA	0.23	(0.03; 0.44)	0.51	0.030	0.26	0.13	(−0.10; 0.36)	0.28			0.18	(−0.47; 0.82)	0.29							
MBT-SO	0.32	(−0.09; 0.74)	0.38	0.118	0.15	−0.10	(−0.57; 0.36)	−0.12			−0.87	(−2.39; 0.66)	−1.02							
MBT-G	0.21	(−0.07; 0.49)	0.37	0.132	0.14	−0.09	(−0.40; 0.23)	−0.15			0.12	(−0.50; 0.73)	0.20							
BBS	0.09	(−0.02; 0.21)	0.41	0.092	0.17						−0.18	(−0.55; 0.19)	−0.78							
SFT-1	0.09	(−0.04; 0.22)	0.35	0.155	0.12	0.04	(−0.18; 0.26)	0.16	0.250	0.18	−0.29	(−0.81; 0.24)	−1.11	0.311						
SFT-2	0.12	(0.02; 0.22)	0.52	0.027	0.27	0.08	(−0.06; 0.23)	0.36			0.17	(−0.06; 0.41)	0.75							
SFT-3	0.00	(−0.04; 0.04)	−0.05	0.851	0.00	0.00	(−0.05; 0.06)	0.04			0.03	(−0.06; 0.13)	0.46							
SFT-4	0.02	(−0.01; 0.05)	0.30	0.233	0.09	0.03	(−0.01; 0.06)	0.39			0.03	(−0.03; 0.09)	0.42							
SFT-5	0.00	(−0.01; 0.00)	−0.22	0.373	0.05	0.00	(−0.00; 0.00)	−0.26			0.00	(−0.01; 0.02)	0.50							
SFT-6	−0.13	(−0.37; 0.10)	−0.29	0.246	0.08	−0.12	(−0.52; 0.28)	−0.27			−0.37	(−1.33; 0.60)	−0.80							
MMSE	0.08	(−0.00; 0.16)	0.46	0.055	0.21						−0.07	(−0.26; 0.12)	−0.40							

Dependent variable: ICAP. ICAP: Inventory for Client and Agency Planning; MBT-T: Mini BESTest total; MBT-APA: Mini BESTest anticipatory postural adjustments; MBT-CPA: Mini BESTest reactive or consecutive postural adjustments; MBT-SO: Mini BESTest sensory orientation; MBT-G: Mini BESTest gait; BBS: Berg Balance Scale; SFT-1: Chair Stand Test; SFT-2: Arm Curl Test; SFT-3: Chair sit and reach test; SFT-4: Back scratch test; SFT-5: 6 min walk test; SFT-6: 8 foot up and go test; MMSE: Mini Mental State Examination.

## Data Availability

The data presented in this study are available on request from the corresponding author.
